# Pattern Formation upon Evaporation of Sessile Droplets of Polyelectrolyte/Surfactant Mixtures on Silicon Wafers

**DOI:** 10.3390/ijms22157953

**Published:** 2021-07-26

**Authors:** Lionel Perrin, Andrew Akanno, Eduardo Guzman, Francisco Ortega, Ramon G. Rubio

**Affiliations:** 1 Departamento de Química Física, Facultad de Ciencias Químicas, Universidad Complutense, Ciudad Universitaria s/n, 28040 Madrid, Spain; drewakanno@gmail.com (A.A.); eduardogs@quim.ucm.es (E.G.); fortega@quim.ucm.es (F.O.); 2 Institute Lumière Matière, Claude Bernard University Lyon 1, Bâtiment Alfred Kastler—4ème Etage Domaine Scientifique de La Doua, 10 Rue Ada Byron, CEDEX, 69622 Villeurbanne, France; 3 Instituto Pluridisciplinar, Universidad Complutense, Paseo Juan XXIII 1, 28040 Madrid, Spain

**Keywords:** evaporation, salt, surfactant, sessile droplet, patterning, Marangoni flow

## Abstract

The formation of coffee-ring deposits upon evaporation of sessile droplets containing mixtures of poly(diallyldimethylammonium chloride) (PDADMAC) and two different anionic surfactants were studied. This process is driven by the Marangoni stresses resulting from the formation of surface-active polyelectrolyte–surfactant complexes in solution and the salt arising from the release of counterions. The morphologies of the deposits appear to be dependent on the surfactant concentration, independent of their chemical nature, and consist of a peripheral coffee ring composed of PDADMAC and PDADMAC–surfactant complexes, and a secondary region of dendrite-like structures of pure NaCl at the interior of the residue formed at the end of the evaporation. This is compatible with a hydrodynamic flow associated with the Marangoni stress from the apex of the drop to the three-phase contact line for those cases in which the concentration of the complexes dominates the surface tension, whereas it is reversed when most of the PDADMAC and the complexes have been deposited at the rim and the bulk contains mainly salt.

## 1. Introduction

Contact angle, spreading, and evaporation phenomena are ubiquitous in nature and in technological processes, such as heat exchanges, ink-jet printing, pesticide applications, cosmetics and pharmacology, the food industry, etc. Despite the fact that the study of contact angle has attracted the attention of scientists since the 19th century, it still is an active field of research because new experimental and computational possibilities have revealed subtle details that had not been considered in the early times [1,2].

**Figure 1:**
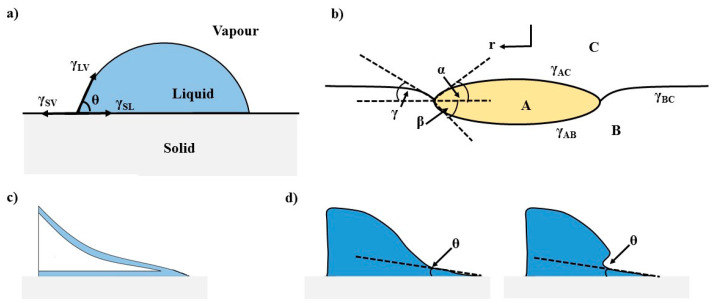
(**a**) Contact angle (*θ*) and interfacial tensions for an axisymmetric dropped onto a solid substrate. (**b**) Contact angles and interfacial tensions for an axisymmetric dropped onto a liquid surface (liquid lens), where A and B represent two liquid phases and C the third fluid (vapor or liquid)**.** (**c**) Sketch of the liquid profile close to the three-phase contact line at a mesoscopic scale. (**d**) Sketch showing details of the real shape of the drop near the three-phase contact line of the liquid/solid/vapor interface at a mesoscopic scale.

A simplified approach to the problem is sketched in Figure 1, where an axisymmetric fluid droplet is in equilibrium with two immiscible phases, e.g., solid and vapor or liquid and vapor, defining a three-phase contact line (TPCL). Either a thermodynamic or a mechanical approach to the problem can lead to the well-known Young equation

(1) $$\gamma_{\mathrm{sv}}-\gamma_{\mathrm{sl}}=\gamma_{\mathrm{lv}}\cos\theta ,$$

where *γ*_sv_, *γ*_sl_, and *γ*_lv_ are the interfacial tensions of the solid/vapor, solid/liquid, and liid/vapor interfaces, respectively, and *θ* is the so-called equilibrium contact angle.

It should be noted that the classical approach to the problem, though most frequently used for technological purposes, was developed for a macroscopic fluid droplet onto a perfectly smooth solid, and neglected the detailed physics dominating the behavior of the droplet near the TPCL. It must be considered that there are transitions between the bulk, where intermolecular potentials and hydrodynamics dominate the properties. Furthermore, the double layers of the liquid/vapor and solid/vapor interfaces are very far apart, to the region where they start to overlap, and Derjaguin forces start to dominate the behavior (see Figure 1c). Finally, a vapor layer adsorbed onto the solid, where statistical mechanics have to be considered. The continuity between the three regions above mentioned can lead to a complicated shape near the TPCL, as illustrated in Figure 1d. The inclusion of the Derjaguin forces is enough to explain the existence of the contact angle hysteresis (Δ*θ*) usually observed in the experiments, even for very smooth surfaces. Δ*θ* is defined as the difference between the advancing and the receding contact angles, Δ*θ* = *θ*_a_ − *θ*_r_, and can take values of several degrees [3].

Δ*θ* is crucial, both in theory and in practical applications, because when a drop is deposited on a substrate, two phenomena usually take place: spreading and evaporation. Both are non-equilibrium processes that are accompanied by hydrodynamic flows derived from the creation of Marangoni stresses [4]. If the substrates are not perfectly smooth, pinning and depinning phenomena complicate the situation, and even the loss of the circular shape of the TPCL can happen, as observed in patterned substrates [5].

In practical applications, mixtures instead of pure liquids are commonly used, which leads to time-dependent interfacial tensions associated with a differential adsorption of the components at the interfaces [4,6]. This leads to surface tension gradients which originate Marangoni stresses during the evaporation process. This emerges as a very interesting aspect from a technological perspective because many applications rely on the removal of the solvent excess from droplets of colloidal suspensions leading to deposits of solutes onto substrates whose surfaces are usually rough [7]. During evaporation, the droplet contact line can become pinned at a defect of the substrate surface for a significant period of time due to the balance between capillary-pressure and disjoining-pressure gradients. The pinning of the contact line accelerates droplet solidification, leading to particle deposition near the droplet edge which leads to the formation of a coffee-ring pattern [8,9,10]. Several recent reviews on the topic have been published [8,11,12]. In the case of mixtures with one or several non-volatile components, the advance of the evaporation pushes the mixtures beyond the saturation concentration of one or several components. This leads to precipitation and the so-called “coffee-ring” effect. It must be stressed that nature takes advantage of these interfacial phenomena, leading to striking behaviors such as those of superhydrophobic, superhydrophilic, and superomniphobic surfaces [13].

The absence of contact line pinning leads to the appearance of deposits with different morphologies, including concentric rings [14] and conical patterns [15]. In other studies, successive pinning and depinning of the contact line on the substrate surface has also been observed [16]. Among the most common patterns, ring-like shapes, bumps, multiple concentric rings, uniform deposits, or hexagonal arrays are included [17,18,19,20,21]. The suppression of the ring-shaped residue pattern is very useful for different technological applications, e.g., spray coating [22], microfabrication [23], bioassays [24,25], optoelectronic device manufacturing [26], DNA microchip fabrication [27,28], and production of solar energy absorption devices [29]. The patterns formed from drying biological fluids might be used for simple and rapid disease diagnosis [30]. Some empirical relationships between the patterns of dried droplets and different diseases have been established: carcinoma, viral hepatitis type B, Waldenstrom’s disease, burn disease, tuberculosis, and leprosy [24,31,32]. It should be noted that even though the coffee-ring effect presents a high potential in many technological applications, it is deemed detrimental in many other fields, e.g., production of coatings [33] or detection of biomolecules using different spectroscopies [34]. The above discussion points out that the study of the behavior of a fluid drop onto a solid surface and in contact with its vapor is an intricate problem where equilibrium and non-equilibrium concepts cannot be separated. Therefore, the understanding of the physico-chemical aspects underlying the formation of the coffee-ring patterns upon the drying of droplets containing colloidal objects presents a paramount interest for both academia and industry.

This work describes the morphology and chemical composition of the coffee-ring-like deposits appearing on hydrophilic silicon dioxide surfaces upon the complete evaporation of a sessile droplets containing polyelectrolyte–surfactant mixtures. These mixtures are commonly included in shampoo–conditioner formulations, and hence their study presents key importance for the cosmetic industry, although the solid substrates used in this work are far from the complexity of real hair fibers, where the pinning effects will be stronger than in our substrates. It must be remarked that the performance of these formulations is related to the layer of polyelectrolyte–surfactant complexes adsorbed on the hair fibers after drying [35,36]. The optimization of the formulations makes it necessary to know the morphology of the deposit in order to minimize its friction coefficient. This work discusses the behavior of mixtures formed by poly(diallyldimethylammonium chloride) (PDADMAC) as polycation and two different anionic surfactants, sodium laureth sulfate (SLES) and sodium N-lauroyl-N-methyltaurate (SLMT). It should be noted that beyond the cosmetic industry, polyelectrolyte–surfactant mixtures present a broad interest in many other industries, e.g., detergency, tertiary oil recovery, production of dietary and pharmaceutical products, or wood pulping [35,37,38]. Therefore, the understanding of the pattern formation on surfaces upon the complete evaporation of aqueous solutions containing polyelectrolyte-mixtures is a very complex problem with a broad technological interest.

## 2. Results

The analysis of the deposits onto the hydrophilic silicon dioxide surfaces obtained after drying of droplets (3–4 µL), at 25 °C and relative humidity 30%, was performed on the bases of SEM images and the elemental analysis of the resulting patterns. It is worth noting that the mixtures of PDADMAC and the anionic surfactants (SLES or SLMT) were composed of positively charged PDADMAC–SLES or PDADMAC–SLMT complexes obtained upon the binding of the anionic surfactant to the polycation, which drives the release of the corresponding counterions (Na^+^, Cl^−^) resulting in a large entropy increase, as discussed in previous publications [39,40]. Further details on the characterization of the association and the interfacial properties related to the water/vapor and water/solid surfaces are discussed in our previous publications [40,41].

### 2.1. SImages

**Figure 2:**
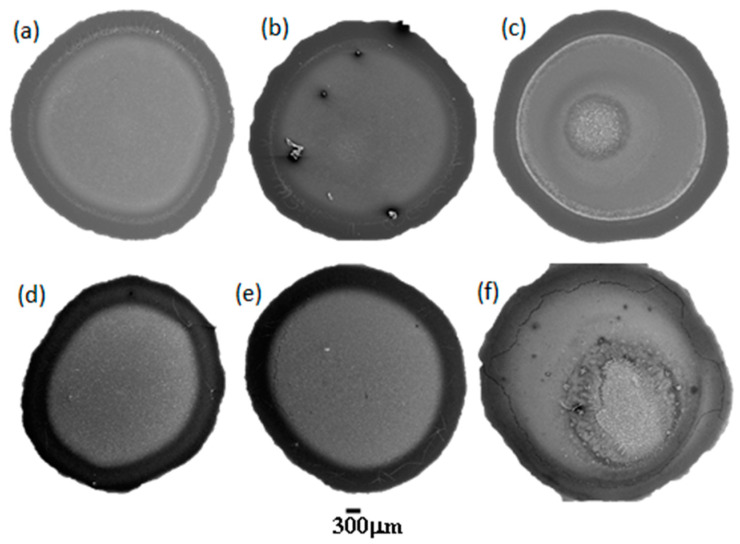
SEM images of dried droplets of PDADMAC–SLES mixtures with SLES concentrations of (**a**) 26 μM, (**b**) 0.26 mM, and (**c**) 2.6 mM, and of PDADMAC–SLMT mixtures with SLMT concentrations of (**d**) 2.9 μM, (**e**) 29 μM, and (**f**) 2.9 mM.

The SEM images of dried droplets for PDADMAC–SLES and PDADMAC–SLMT mixtures with different surfactant concentrations are shown in Figure 2. These images show the formation of patterns with similar morphology independently of the specific nature of the polyelectrolyte–surfactant mixture, and the variation of the structure of the deposits with surfactant concentration. The deposits cover the entire wetted area and their morphologies are consistent with the formation of coffee-ring-like patterns [42,43]. The morphologies are composed of a peripheral ring and a uniform distribution of crystals that starts from the rim and covers the entire pattern. The increase of the surfactant concentration leads to the emergence of a distinct crystalline structure that starts to accumulate in the center of the deposit. It must be stressed that the EDS spectra (discussed later) reveals that the entire surface of the pattern is covered by a thin layer of polyelectrolyte.

**Figure 3:**
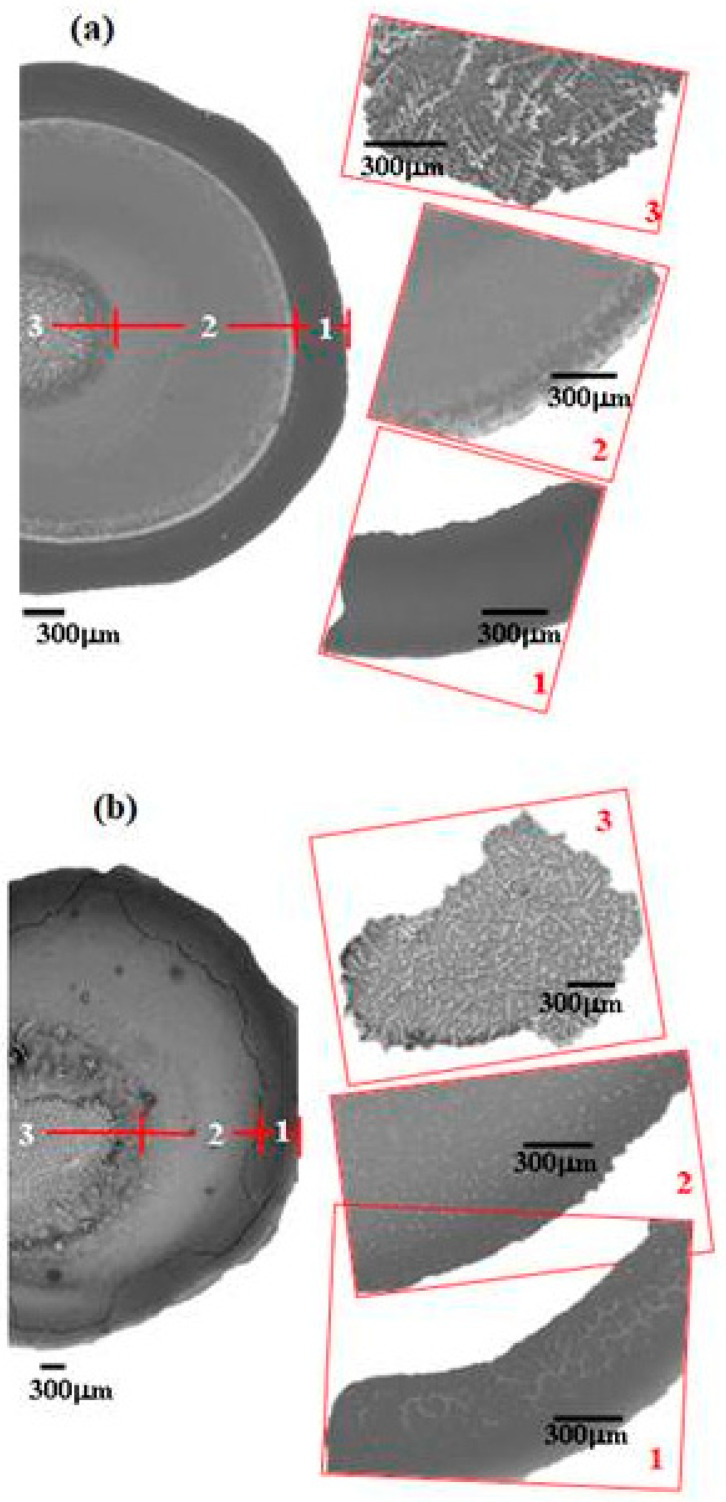
Dried droplets of PDADMAC–SLES (**a**) and PDADMAC–SLMT (**b**) mixtures, with surfactant concentrations of 2.6 and 2.9 mM, respectively, illustrating the three distinct regions.

**Figure 4:**
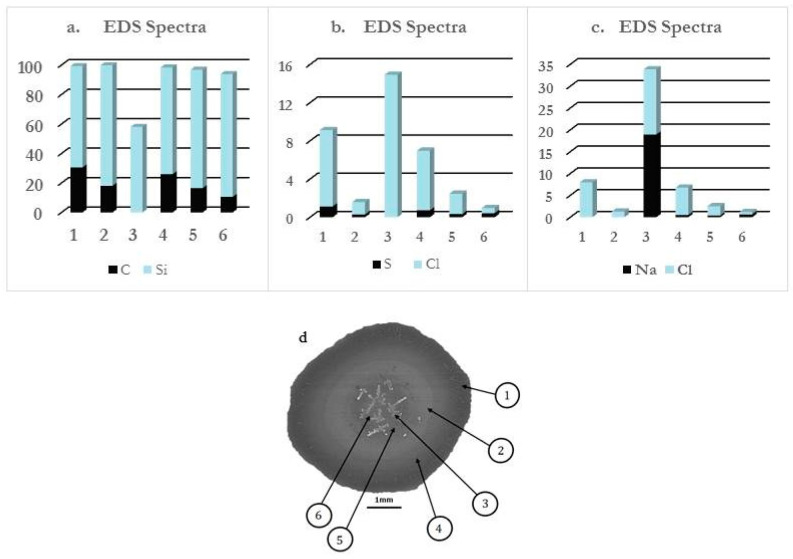
(**a**–**c**) EDS spectra showing the relative amounts of C, Si, S, Cl, and Na on 6 different spots of a deposit of PDADMAC-SLES mixture on a silica wafer. (**d**) SEM image of the deposit showing the 6 different spots. NB: spot 3 indicates the penetration of the EDS beam through the salt crystal.

A detailed analysis of the patterns allows one to distinguish three different regions (“Region 1”, “Region 2”, and “Region 3”) as is illustrated in Figure 3 for dried droplets of both mixtures. The combination of the SEM images corresponding to the deposits with their respective EDS spectra allows obtaining a better understanding of the distribution of the different species within the formed pattern (see Figure 4 for an example of the results obtained for one of the samples).

On the basis of the obtained results is possible to summarize the following characteristics for the obtained deposits:

- Region 1 is the perimeter of the pattern, i.e., the coffee ring which appears in all of the deposits, in agreement with those reported for dried droplets of other complex mixtures [43,44]. The obtained results suggest that the thickness of the ring is independent of the surfactant concentration, which might be ascribed to the similar composition of the ring in all cases. This may be understood considering that the formation of the ring is associated with the presence of PDADMAC in the mixture which presents a constant concentration (5 g/L). In addition, cracks, associated with the release of internal stresses which accumulate during the drying process, are observed in the ring areas which are more obvious when PDADMAC–SLMT deposits are considered.
- Region 2 is the secondary area of the deposits which is made up of crystal-like structures distributed uniformly within the droplet. These structures are NaCl crystals formed as a result of the release of counterions during the binding of the anionic surfactant to the polycation. The formation of this salt crystals agrees with the picture expected from the binding isotherm reported in our previous publication [40], with their deposition and growth being probably related to an anisotropy of surface tension between the substrate and the droplet mixture [45]. This may lead to a concentration gradient that favors the growth of the salt crystals, with such growth being commonly oriented towards the external polymeric ring. The results show that the salt crystals start to be formed from the edge of the pattern, appearing uniformly distributed close to the center of the droplets. It should be stressed the studied mixtures do not contain inorganic electrolytes, and hence the salt crystals can be only ascribed to the release of counterions occurring during the association between the PDADMAC and the surfactant. This contrasts with the salt crystal found in deposits obtained upon evaporation of other complex mixtures in which inorganic electrolytes were initially added [45,46].
- Region 3 is located at the center of the obtained deposits, emerging as an accumulation of crystal structures.

It should be noted that the crystalline structures forming Region 3 are only observed at the highest surfactant concentration studied. The salt crystals are larger than those found in Region 2, which might be related to the direction of crystal growth observed in Region 2. This is plausible considering that the evaporation rate is similar for all the mixtures, and that the formation of these patterns emerges dependent on the drying rate [46,47]. Furthermore, the existence of Region 2 in all dried droplets indicates that the mechanism of pattern formation is similar, at least during the first part of the evaporation process, with independence of the surfactant concentration.

It should be stressed that the morphology of the deposits depends on whether d*γ*/d*c*, i.e., the slope of the surface tension isotherm corresponding to the adsorption of a surface-active molecule at the water/vapor interface, is positive or negative [48]. This was confirmed by Hu and Larsson [49] in their study about the evaporation of a polymer solution. They found the formation of volcano-like structures at the center of the deposit. Very recently, Efstratiou et al. [50] found the formation of a rim of evenly spaced salt crystals instead of a continuous rim after the evaporation of aqueous saline droplets. Furthermore, they also found that the type of salt, its initial concentration, and the hydrophilic or hydrophobic character of the substrate can drive the formation of salt deposits with very different morphologies, e.g., continuous rings, concentric rings, or discrete crystals, close to the ring or at the center of the deposit. It is worth mentioning that the evaporation of saline solutions is slowed down with respect to water because of the well-known decrease of the vapor pressure [51], which decreases the water vapor concentration far from the droplet surface.

**Figure 5:**
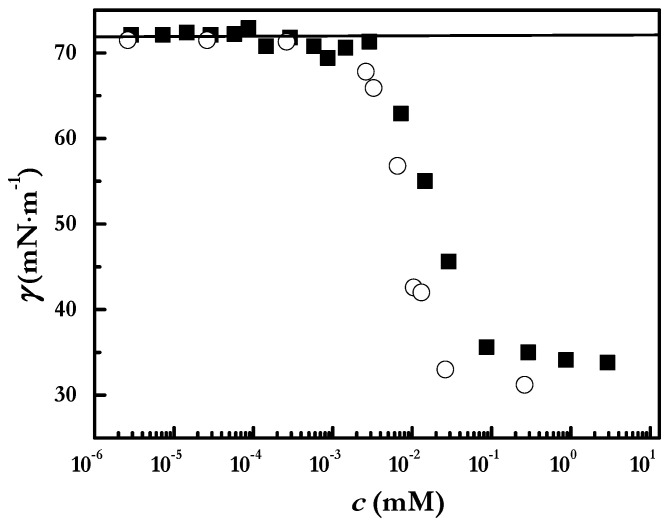
Surface tension isotherms as were obtained using a drop shape tensiometer for PDADMAC–surfactant solutions with a fixed polymer concentration of 5 g/L: PDADMAC–SLMT (■) and PDADMAC–SLES (○). The solid lines represent the surface tension of the pure Milli-Q water used in this work. Adapted from Akanno et al. [6], with permission under attribution license Creative Commons 4.0 (2021).

To understand the accumulation of NaCl at the center of the deposit, the different sign of d*γ*/d*c* and polyelectrolyte–surfactant mixtures must be taken into account. Thus, for salt solutions d*γ*/d*c* > 0, whereas the sign is reversed (d*γ*/d*c* < 0) when polyelectrolyte–surfactant mixtures of intermediate concentrations are concerned, and assumes a quasi-null value (d*γ*/d*c* ~ 0) value for mixtures with very low and very high surfactant. This picture is clear from the interfacial tension-surfactant concentration isotherms displayed in Figure 5 (further discussion on the specific dependences of the surface tension on the surfactant concentration for polyelectrolyte–surfactant solutions, and their differences with the isotherms for the surfactant solutions can be found in our previous publication [40]). The above-discussed differences in d*γ*/d*c* provide a justification for the tendency of the Marangoni flow to move the polymer–surfactant complexes towards the three-phase contact line at the beginning of the drop evaporation when the salt concentration is relatively small. This is possible because the instantaneous volume of the droplets *V* is not much smaller than their initial volume *V*_0_ as was demonstrated in our previous paper [6]. Despite that the d*γ*/d*c* is very small, thus leading to values of *γ* ≈ *γ*_0_ (with *γ*_0_ being surface tension of water) for the initial salt solution, the salt concentration becomes very high during the latter stage of evaporation, so that *γ* is several mN/m^−1^ higher than *γ*_0_. Furthermore, as was stated in our previous publication the *γ* vs. surfactant concentration curves for the polymer–surfactant mixtures have the same shape as that of the surfactant solutions [40], and it is expected that d*γ*/d*c* ≈ 0 for high surfactant concentrations as the evaporation proceeds. For the sake of example, for the lowest initial surfactant concentration (2.6 μM), the surfactant concentration, 26 μM at which γ becomes constant is reached after an evaporation time *t* = 750 s [6]. At this time the only contribution to the Marangoni flow is that associated with the NaCl formed upon the binding of surfactant molecules to PDADMAC chains. The formation of salt deposits and the reversal of the coffee ring have been described in the last decades by other authors [44,49,52,53].

### 2.2. Governing Mechanism for Crystal Formation and Crystal Size Variation with Surfactant Concentration

**Figure 6:**
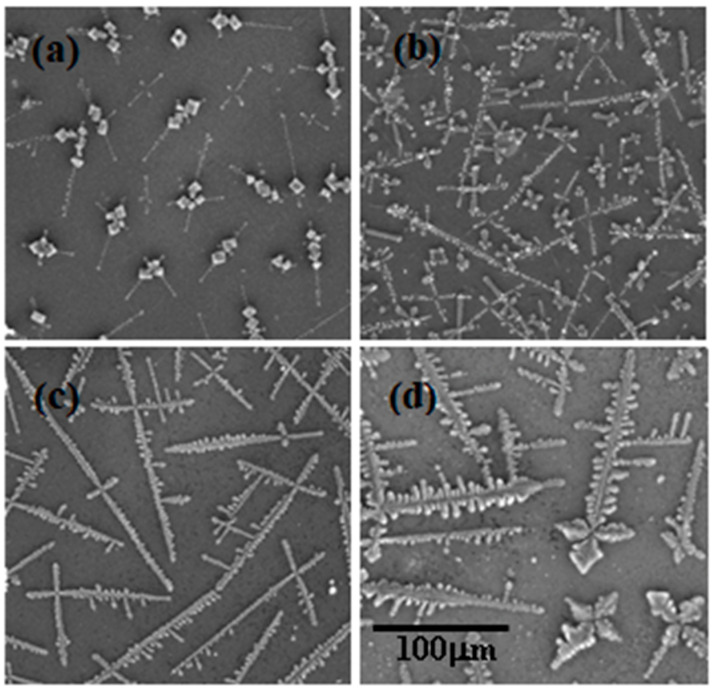
SEM images of dried droplets of PDADMAC–SLMT mixtures depicting the variation in NaCl crystal size with SLMT concentrations of: (**a**) 2.9 μM, (**b**) 29 μM, (**c**) 0.29 mM, and (**d**) 2.9 mM.

The salt crystals formed on evaporation of the mixture droplets were observed to be dendrite-like structures as is shown in Figure 6. The physical mechanism governing self-forming dendrites from solutions have been studied extensively by Langer [54] on the basis of the Mullins–Sekerka instability concept, which accounts for the formation of dendrites with side branching. Mullins and Sekerka [55] explained that for a diffusion-controlled crystal growth, any instability in the system will affect the concentration gradients and favor the growth of the crystal.

The stability of a diffusion-controlled crystallization process of a solution on a solid surface is related to the gradient of the solute concentrations between the solid surface and the bulk solution and a capillary effect due to the surface tension at the solid–solution interface. Mullins and Sekerka [55] showed that the growth of the solute crystals on the solid surface is the result of some perturbations within the system, which are associated with a local solute concentration gradient. Furthermore, such a concentration gradient is also related to the solid–solution surface tension, and hence the variation of this quantity at the solid–liquid interface may enhance the importance of the instabilities.

**Figure 7:**
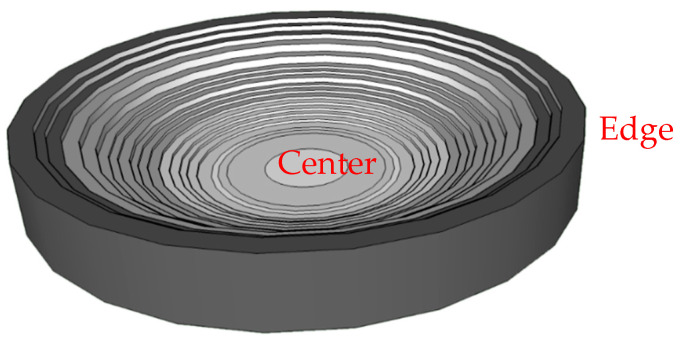
Idealized picture of the variation in thickness of the pattern formed by the evaporated droplet of the polyelectrolyte–surfactant mixture. The thickness of the deposit decreases from the edge (darker region) to the center (lighter region).

In the case of evaporating sessile droplets of polyelectrolyte–surfactant mixtures, the existence of temperature gradients between the surface of the sessile droplet and the silicon wafer is an indication that the drying process is both thermal and diffusion-driven. Additionally, in the solid–liquid equilibrium, the salt concentration in the droplet is much greater than at the silicon wafer surface, thus NaCl molecules are constantly ejected to the solid surface in a similar way that latent heat is released from a melt during solidification [56], and the interfacial salt composition grows with time. On the other hand, the deposition of the positively charged complexes on the negatively charged silicon wafer surface may result in a charge inversion of the substrate surface [57]. The elemental analyses (EDS spectra displayed in Figure 4) of the patterns also reveal an increase in the height of the spectrum of silicon from the droplet rim to the central region which could mean that the thickness of the pattern formed on the silicon wafer decreases in the same direction as sketched in Figure 7. Therefore, there is the possibility that a gradient of charge distribution exists on the surface of the pattern from the more positively charged edge to the less positively charged central region. This will create a gradient of surface energy on the silicon wafer surface eventually leading to variations of the solid–liquid interfacial tension, which is likely the source of the interfacial instabilities in the case of the evaporating droplets of the mixtures in the current study.

**Figure 8:**
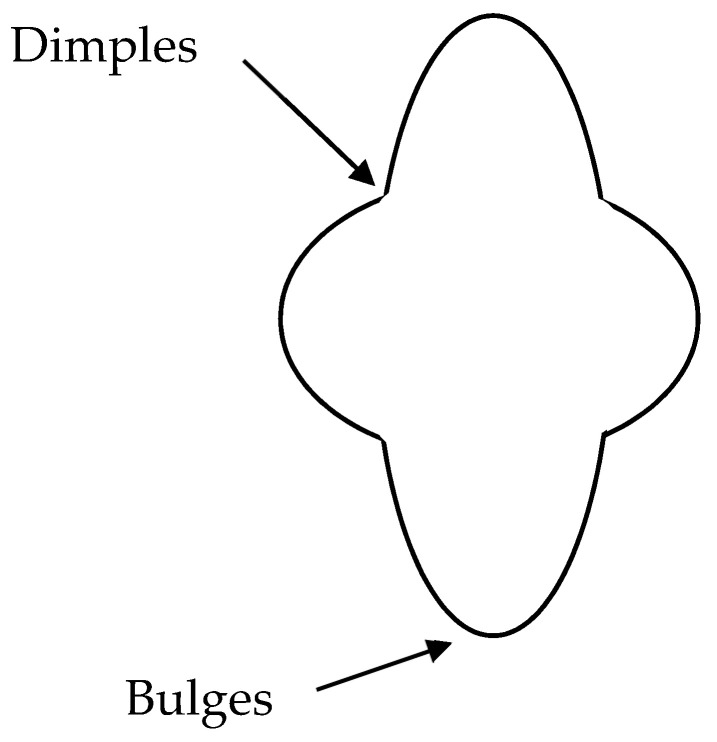
Idealized picture representing the perturbed state of an initially spherical particle under the influence of interfacial instabilities.

Mullins and Sekerka [55] stated that the instabilities would create a gradient in the interfacial salt concentration producing bulges and dimples on an initially growing spherical particle as is schematized in Figure 8. The increase of the perturbations in the system promotes the growth of the salt crystals observed in the patterns. Interestingly, these interfacial instabilities are unaffected by the solute concentration in the bulk solution [57], and therefore salt crystals were observed in the patterns formed for all the surfactant concentrations. Furthermore, the structures presented in the current study consist of a main branch with a length of hundreds of microns connected to shorter branches on the sides. Additionally, the side branches vary in size and appear to be equally spaced on the main branch. Furthermore, the size of these dendrites increases with the surfactant concentration, which can be linked to an increase in the concentration of the counterions in the mixture. It should be noted that the concentrations of Na^+^ and Cl^−^ in the solution are very far from the saturation concentration of aqueous NaCl solution (6.15 M) [58], and hence it may be expected that only close to the complete evaporation of the droplets do well-defined dendritic-like crystals appear on the silicon wafer surface. This increase may be only apparent because, as was already mentioned, Na^+^ and Cl^−^ ions are also present in the rim of the deposit, although no quantitative analysis of the real salt concentration can be done with the present data. Moreover, it cannot be forgotten that when NaCl crystals start to appear, tiny amounts of surfactant and polymer may still be present in the solution. The high solubility of NaCl in water provides a justification for the increase of the size of the dendritic-like structures with the surfactant concentration. Thus, the higher the surfactant concentration, the easier the depletion of the NaCl from the aqueous solution, and hence the growth of dendritic-like structures appears favored.

In addition to the presence of interfacial instabilities at the solid–liquid interface promoting the growth of crystal structures on the solid surface, Coriell and Sekerka [59] pointed out that the existence of anisotropy in the solid–liquid interfacial tension will cause these instabilities to be direction dependent. The crystals observed in the patterns formed upon the evaporation of droplets of PDADMAC–SLES and PDADMAC–SLMT mixtures were randomly distributed. This leads to well-defined directional growth which agrees with the existence of anisotropy in surface tension at the solid-liquid interface within the droplet of the mixture and the PDADMAC-modified substrate surface. Finally, it must be taken into account that, once crystallization starts, a local concentration gradient is created because in the immediate vicinity of the crystal surface the solution is supersaturated and at a very short distance it is saturated. Such gradient creates an associated surface tension gradient that establishes a jet-like flow towards the growing crystal edge (recall that when crystallization takes place, there is almost only salt in the solution, thus dγ/d*c* > 0).

## 3. Materials and Methods

### 3.1. Chemicals

PDADMAC with a weight average molecular mass *M*_w_ in the 100–200 kDa range was purchased as a 200 g/L aqueous solution from Sigma-Aldrich (St. Louis, MO, USA) and was used as received. The anionic surfactant SLES, with an average number of 2 oxyethylene, was supplied by Kao Chemical Europe S.L. (Barcelona, Spain) as an aqueous solution of 70 wt% of surfactant concentration. It was purified by lyophilization followed by recrystallization of the obtained powder using acetone for HPLC (Acros Organics, Morris Plains, NJ, USA) [60]. SLMT was synthesized and purified following the procedure described in our previous publication [39].

Ultrapure deionized water used for cleaning and solution preparation was obtained by a multicartridge purification system aquaMAX^TM^-Ultra 370 Series (Young Lin Instrument, Co., Anyang, Korea). The water used had a resistivity higher than 18 MΩ∙cm, and a total organic content lower than 6 ppm.

### 3.2. Preparation of the Polyelectrolyte–Surfactant Solutions

Aqueous solutions of polyelectrolyte–surfactant mixtures, containing a fixed PDADMAC concentration of 5 g/L and different surfactant concentrations in the range of 10^−6^–5 mM were used. The preparation of the aqueous mixtures was performed according to the procedure reported in our previous work [39]. This procedure can be briefly summarized in the following steps: (i) the required amount of the aqueous commercial solution of PDADMAC for obtaining final mixtures with a polymer concentration of 5 g/L was weighed and poured into a flask; (ii) surfactant was added to the flask containing the polyelectrolyte. For this purpose, surfactant solutions (pH ~ 5.6) with concentrations one order of magnitude higher than the surfactant concentration required in the final mixture were weighted and added to the flasks containing PDADMAC. (iii) The mixture was diluted with an acetic acid solution of pH ~ 5.6 to the final bulk composition. During the mixture preparation, there was no delay between the addition of the different components. The final mixtures were homogenized by mild stirring at 1000 rpm using a magnetic stirrer for 1 h at room temperature, and then they were left to age for 1 week prior to use. This aging period was used to ensure that no turbidity appeared in the mixtures and that the transparency of the mixtures after preparation was maintained [39]. The above procedure has been shown to demonstrate a good level of reproducibility for obtaining kinetically arrested states after mixing the PDADMAC and surfactant [39].

The pH of all the solutions was fixed at 5.6 using glacial acetic acid (purity > 99%). It should be noted that the pH, the PDADMAC concentration, and the use of acetic acid for fixing the pH were not arbitrary choices. Such conditions were adopted to mimic the characteristics of hair-conditioning formulations used for reducing the bleaching of hair fibers under application conditions [61,62].

### 3.3. Preparation of the Silicon Wafer Surface

Silicon wafers 1 inch in diameter were supplied by Siltronix (Archamps, France) and were cleaned, just before their use, by immersion in piranha solution (mixture of sulfuric acid and hydrogen peroxide in a volume ratio 7:3) for 30 min, followed by rinsing with Milli-Q water and absolute ethanol. Afterwards, the wafers were dried under a nitrogen stream. This procedure resulted in the formation of a homogeneous layer of hydrophilic silicon dioxide on the wafer surface, with a thickness in the range 2–3 nm as was determined by ellipsometry.

### 3.4. Scanning Electron Microscope

The morphologies of the patterns formed on the surfaces after evaporation of the mixtures were analyzed using scanning electron microscopy (SEM). Two different Scanning electron microscopes, with magnification up to 5000×, from JEOL (models JSM 6400 and JSM 6335F-Akhisima, Japan) were used for studying the morphologies and structures of the obtained deposits. The JSM 6400 operates with a beam voltage of 25 kV and presents image resolution of up to 10 nm, whereas the JSM 6335F operates at a lower beam voltage (15 kV), enhancing the resolution up to 1.5 nm. Furthermore, the latter is coupled to an energy-dispersive X-ray spectrometer (EDS) allowing us to perform the elemental analysis of the deposits.

### 3.5. Surface Tension Measurements

The dependences of the equilibrium interfacial tension *γ* on the surfactant concentration upon adsorption of polyelectrolyte–surfactant aqueous solutions to the water/vapor interface was followed using a home-made profile analysis tensiometer in pendant drop configuration (for further details see reference [39]). The adsorption at the water/vapor interface was measured until a steady state was reached, i.e., changes of surface tension smaller than 0.1 mN·m^−1^ during 30 min. Special care was taken to minimize the evaporation effects during these experiments.

## 4. Conclusions

The patterns formed on the silicon wafers upon the complete evaporation of sessile droplets of polyelectrolyte–surfactant mixtures present two well-differentiated regions, with the appearance of a third one for mixtures with high surfactant concentration. The formation of patterns on the substrate leads to a pinning of the contact line to the substrate as result of the deposition of PDADMAC at the periphery of the droplet, which is strongly favored due to the electrostatic interactions at the PDADMAC-surface. The relatively constant size of the ring is an indication that the coffee ring is mainly due to the PDADMAC in the mixture and independent of the size of the PDADMAC–surfactant complexes formed in the mixture. Furthermore, the appearance of cracks around the coffee ring, associated with the release of internal stress that accumulates during evaporation, is observed. Capillary and solutal Marangoni flows drive the deposition of PDADMAC molecules and PDADMAC–surfactant complexes at the periphery of the deposits as the evaporation proceeds, with the resulting pattern depending on the surfactant concentration, even though no influence of the nature of the surfactant was found. The peripheral zone is followed by a secondary region composed of dendrite-like structures of NaCl uniformly distributed in the interior of the pattern. These NaCl crystals are the result of precipitation of the counterions released during the binding of the surfactant to the polyelectrolyte chains in a salt-free mixture, with their size increasing with surfactant concentration. The deposition of salt crystals in salt-free solutions should be considered a new physico-chemical scenario because previous studies dealing with salt crystal formation after dying were concerned to systems in which inorganic electrolytes were initially contained in the solution.

The formation of the dendrite-like structures may result from the instabilities produced due to surface tension anisotropies which are promoted throughout the interior of the droplet of the mixtures. This instability appears after PDADMAC–surfactant complex deposition. Furthermore, the motion of the counterions towards the center of the drop leads to a uniform distribution of the salt crystals in the interior of the ring. At the highest surfactant concentration, the excess concentration of counterions produced in the mixtures was observed to precipitate and accumulate around the center of the droplet forming a third region of large dendrite structures.

The final patterns result from two different Marangoni flows which present different circulation. This may be rationalized in terms of the different d*γ*/d*c* existing for polyelectrolyte–surfactant complexes and for counterions. To the best of our knowledge, this work reports the first evidence of such type of phenomena. Furthermore, another interesting aspect of the crystal formation in the current study is that they are self-growing and only appear in the presence of the polyelectrolyte. Presumably, the accumulation of PDADMAC–surfactant complexes at the contact line provides the suitable environment for crystallization of the salt as no crystal structure was observed with a salt solution of the same concentration as the counterions in the mixtures.

The understanding of the here-contained aspects presents a critical impact in the selection of materials for coating applications as these salt crystals might influence the desired properties e.g., strongly modify the spreading properties on the coated surface or its friction coefficient.

## Data Availability

Data are available upon request.

## Abbreviations

*Γ*	equilibrium interfacial tension
*γ*_0_	surface tension of the water
*γ*_sv_	interfacial tension of the solid/vapor interface
*γ*_sl_	interfacial tension of the solid/liquid interface
*γ*_lv_	interfacial tension of the liquid/vapor interface
*Θ*	equilibrium contact angle
*θ*_a_	advancing contact angle
*θ*_r_	receding contact angle
Δ*θ*	contact angle hysteresis
*C*	surfactant concentration
EDS	energy-dispersive X-ray spectrometer
*M*_w_	weight average molecular mass
PDADMAC	poly(diallyldimethylammonium chloride)
SEM	scanning electron microscopy
SLES	sodium laureth sulfate
SLMT	sodium N-lauroyl-N-methyltaurate
*T*	time
TPCL	three-phase contact line
*V*	instantaneous volume of the droplet
*V*_0_	initial volume of the droplet
